# Getting started in computational quantum chemistry

**DOI:** 10.3389/fchem.2013.00014

**Published:** 2013-09-03

**Authors:** Sam P. de Visser

**Affiliations:** School of Chemical Engineering and Analytical Science and Manchester Institute of Biotechnology, The University of ManchesterManchester, UK

**Keywords:** electronic structure theory, molecular structure and function relations, density functional theory (DFT) calculations, quantum theory, computational modeling

In the past few decades, computational resources have become more powerful every year and in addition methodology development has led to much more efficient techniques through parallelization of the calculations and the advent of density functional theory. These reasons make it possible for computational quantum chemists to work on relatively large chemical systems with a total number of atoms well over 100 nowadays. As a result of this, interest in applications of computational quantum chemistry has considerably widened and opened up research opportunities in novel areas. In particular, applications of “realistic” quantum chemical systems have become possible and as such it is starting to become common practise in fields of, e.g., bioinorganic chemistry and biochemistry, to do experimental studies side-by-side with computational modeling. This means that computational quantum chemistry does not operate in virtual worlds and settings anymore on small atomic systems, but can address major chemical problems. These combined experimental/computational studies generally give a broader perspective of a chemical problem and look into it from different angles and perspectives than stand-alone experimental studies. Thus, the computational studies give important additional information alongside experiment and assist in the interpretation of the experimental data. Furthermore, with computational quantum chemistry short-lived catalytic intermediates and their reactivity patterns can be investigated, which coupled to experimental work can explain product distributions and reaction rates. In addition, the computational work can make predictions that encourage future experimental studies. This symbiosis of experiment and theory has led to a large field of research, where theoreticians and experimentalists work together. As a result of that it is not uncommon anymore that PhD students and postdoctoral researchers do a combination of experiment and computation for a single multidisciplinary project. However, although many experimentally based groups are starting to use computational chemistry methods, almost at a routine basis, nowadays there are some serious caveats with the methods and techniques and often these computational studies cannot be done through “black-box”-procedures but require expert supervision.

Although there is an increased popularity of computational quantum chemistry mainly through the use of computational quantum chemistry methods by experimentalists, this does not mean these methods and techniques are routinely done with little or no prior knowledge of the theories and backgrounds. To highlight the difficulties in doing computational quantum chemistry research on experimentally relevant chemical systems, McDouall has written a monograph on the chemical procedures and techniques behind the computational chemistry software packages and the many pitfalls the user should be aware of. The book, therefore, tries to address questions for beginners in doing computational chemistry research, including:

1. What does computational quantum chemistry offer?
2. Where do you start?
3. How do you select a theoretical model?
4. What useful output do I generate and how do I relate this to my experiment?

The book is subdivided into five chapters covering the basics of computational quantum chemistry, electronic structure methods, computation of molecular properties, molecular orbitals, spin densities and relativistic effects. These are the key methods and techniques necessary for computational quantum chemistry in collaboration with experiment and a description of the essential components of the output that can be linked to experiment. Each chapter has a logic set-up that first gives a layman's explanation of the reasons and the background of the basic theories with clear figures. Of course, a quantum chemistry book cannot be complete without equations and there are quite a lot of those in this book. However, these are well explained and McDouall puts them in a broad context and clearly defines their variables and uses. As such I do not feel that the equations scare off the reader here, but are illustrative of the background. There are plenty of examples in the text that explain the theories in better detail.

The book is very well written and is aimed at starters in the field of computational quantum chemistry, such as new Master and PhD students. This book, however, is not like “normal” quantum chemistry books, where the reader gets drowned in very difficult to understand equations that require a high level of Mathematics knowledge. Instead, the author has chosen a selective set of equations and explains in detail what the equation means in chemical terms, what you can do with it and how you can solve the equations. As such, it makes the book highly readable even for starters in the field that not necessarily have a thorough previous background in computational quantum chemistry. It may even be worthwhile for experimentalists who collaborate with computational quantum chemist to read this book and get understanding of what computational quantum chemistry can offer.

What I found particularly useful was the section on converting quantum chemical energies into free energies through thermodynamic state functions and thereby gives experimentally measurable variables. The book is illustrated with a large number of drawings and figures that highlight what is explained in the text.

In summary, the book on “Computational Quantum Chemistry” by McDouall is highly recommended literature for anyone working in the field, collaborating with computational chemists or interested in moving into the field of computational quantum chemistry. The work is very accessible to the lay-reader and should help and assist with getting started in the field.

